# P-304. Scaling Long-Acting Injectable Cabotegravir for PrEP: Adoption, Implementation and Outcomes from a Three-Year Observational Study in a Large Community-Based Clinic Network

**DOI:** 10.1093/ofid/ofaf695.524

**Published:** 2026-01-11

**Authors:** Jessica A Altamirano, Prerak Shukla, Brandon Blankenship, Steven K Barnett

**Affiliations:** CAN Community Health, Miami Gardens, FL; CAN Community Health, Miami Gardens, FL; CAN Community Health, Inc., Saint Petersburg, Florida; CAN Community Health, Miami Gardens, FL

## Abstract

**Background:**

In 2023, only 53% of long-acting cabotegravir (CAB-LA) prescriptions resulted in at least one injection administered at CAN Community Health Network between December 2021 and April 2023. Implementation of Buy-and-Bill began November 2023 with samples available October 2024. We assessed adoption of CAB-LA in the past 3 years and outcomes including rates of initiation, discontinuation, persistence and seroconversion.

**Figure:**
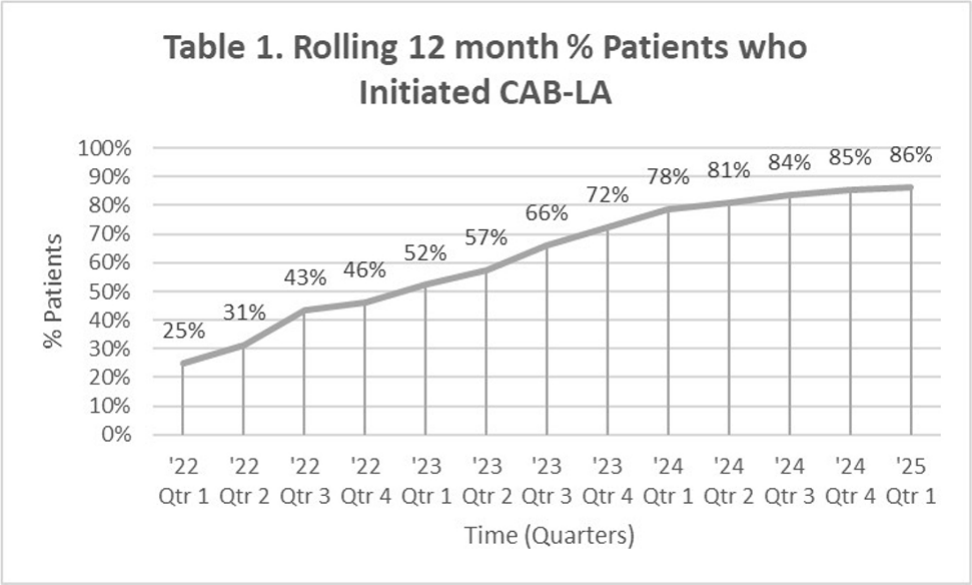

**Figure:**
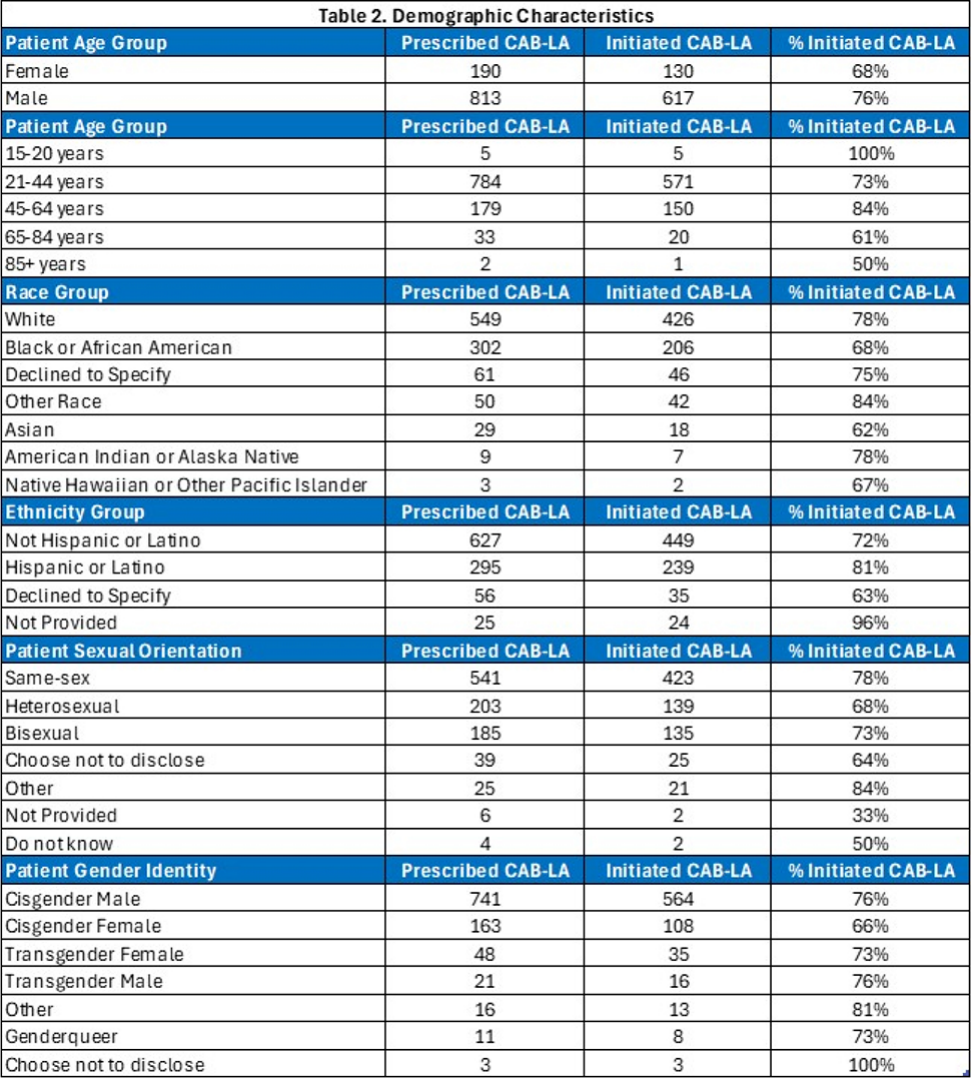

**Methods:**

Power BI Dashboard was built to collect medical data from 24 CAN clinics in 6 U.S. states from December 20, 2021 to March 22, 2025. Initiation rate was defined as proportion of individuals who received ≥ 1 injection of CAB-LA among those prescribed. Persistence was defined by number of injections received. Discontinuation was defined as being > 67 days from last injection or a switch to oral PrEP. HIV seroconversion was defined as positive HIV Ag/Ab test and detectable HIV RNA PCR while receiving on-time injections. PrEP naïve refers to those who did not report PrEP use prior to CAB-LA.

**Figure:**
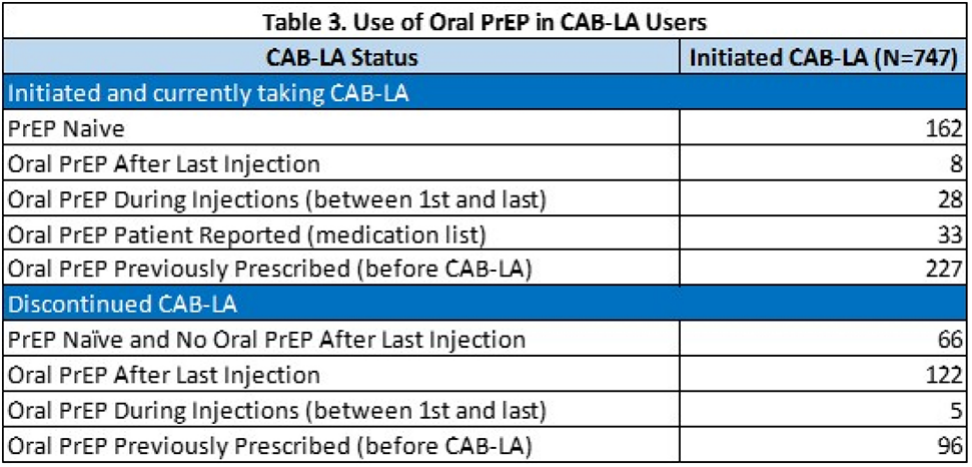

**Results:**

Of the 1,003 HIV-negative individuals prescribed CAB-LA, initiation was seen in 747 (75%) (Table 1); 130 (17%) female and 617 (83%) male sex assigned at birth. 571 were in the age range of 21-44 (76%). Initiation rate within each race group: White (78%), Black or African American (68%), and Asian (62%). Initiation rate in Hispanics was 81% compared to 72% in non-Hispanics. Initiation rate per sexual orientation group was: same-sex (78%), bisexual (73%) and heterosexual (68%). Initiation rate was 77% in transgender males vs. 67% in cisgender females (Table 2). Seroconversion occurred in 2 cases. Median number of injections per person was 6 injections (range 1-19).

Discontinuation was seen in 289 (39%) with 122 switching to oral PrEP (Table 3); median number of 4 injections prior to switch. There were 78 individuals who initiated CAB-LA with a sample; median age was 36. 51 (65%) were cisgender men; 38 (49%) same-sex; 26 (33%) were Black not Hispanic and 44 (56%) were PrEP naïve.

**Conclusion:**

CAB-LA adoption increased over study period, with strong uptake among PrEP naïve users, particularly those starting with samples. Despite 39% discontinuation rate, many switched to oral PrEP with continued engagement in prevention. Seroconversion was rare; disparities by race and gender need further study.

**Disclosures:**

Jessica A. Altamirano, MD, Gilead Sciences: Speaker's Bureau|ViiV Healthcare: Speaker's Bureau

